# Supplementary material for: Targeted gene correction and functional recovery in achondroplasia patient-derived iPSCs
Source: Stem Cell Res Ther. 2021 Aug 28;12:485. doi: 10.1186/s13287-021-02555-8 (PMC8403427; doi:10.1186/s13287-021-02555-8)
Supplement: Supplementary file 1 — Additional file 1. Supplementary material and method - Gene correction of ACH-iPSCs. [file 13287_2021_2555_MOESM1_ESM.docx]

**Supplementary material and method**

**Gene correction of ACH-iPSCs**

1. Design of sgRNAs and ssODNs for ACH-patient

(1) sgRNAs were designed to target point mutation site by using the Guide Design Resouces of Zhang lab (<https://zlab.bio/guide-design-resources>). When the 64 bases in ACH patient sequence centered on the point mutation were input into Guide Design Resouces, dozens of sgRNAs were produced. We selected the one with the highest score, as follows:

sg2RNA-F: caccgatgcaggcatcctcagctac

sg2RNA-R: aaacgtagctgaggatgcctgcatc

(2) ssODNs for homology arm

Taking the mutation point as the center, we used 131 nucleotides (nt) in healthy human *FGFR3* sequence to act as homology arm. The sequence of ssODNs was:

cagccgaggaggagctggtggaggctgacgaggcgggcagtgtgtatgcaggcatcctcagctac**g**gggtgggcttcttcctgttcatcctggtggtggcggctgtgacgctctgccgcctgcgcagcccc.

2. Construction of CRISPR plasmids

The sgRNA2 were synthesized, annealed and ligated to the pSpCas9(BB)-2A-RFP plasmid which was digested with Bbs I (NEB). Single colonies were picked up and performed sequencing using U6 primer.

3. Transfection of CRISPR-Cas9 sgRNA into iPSCs

One million iPSCs were suspended in 100 μl cold Nucleofector solution (Lonza). Thereafter, 5 μg targeting plasmid and 40 μg ssODNs were added into them. The cells were electroporated by using the Human Stem Cell Nucleofector Kit 2 (Lonza) and the Nucleofector 2b Device (Lonza). These cells were seeded into plates by using E8 with ROCK inhibitor. Twenty-four to forty-eight hours after electroporation, about 5000 RFP positive cells were sorted by FACS (BD Aria II) and re-seeded into a 100-mm plate to culture.

4. DNA sequencing analysis

One week later, single cell colonies were picked up and expanded for sequencing analysis. We used SnapGene, SeqBuilder Pro and MegAlign Pro software to conduct sequencing analysis. *FGFR3* primers were:

Forward: 5’-AGGAGCTGGTGGAGGCTGA-3’,

Reverse: 5’-GGAGATCTTGTGCACGGTGG-3’.
