# Supplementary material for: Targeted gene correction and functional recovery in achondroplasia patient-derived iPSCs
Source: Stem Cell Res Ther. 2021 Aug 28;12:485. doi: 10.1186/s13287-021-02555-8 (PMC8403427; doi:10.1186/s13287-021-02555-8)
Supplement: Supplementary file 2 — Additional file 2: Table S1. The sequences of the primers for off-target sites. [file 13287_2021_2555_MOESM2_ESM.docx]

| Gene name | Forward primer | Reverse primer |
| --- | --- | --- |
| *WNT11* | AAGGAATTGGGGAAGCAGGG | GAGGATGAGGATGGTGCGAG |
| *PRAG1* | GCAGCTGCCCCGGCTCATCATCAGCAACT | CCGCTTCATGTCGATCCAGTTGTGCAGCG |
| *GTF2E2* | AGCTGAAGGGCTTCATCGTC | GAACTGCTCCTCTCCTCAGC |
| *FLOT1* | AGCCCATTCGTCTAAACTGGG | TTCCAGTTGCTTCTGTTTTATTAGT |
| *CFAP45* | TCCCTACCATTCTGCGAGGA | ATCCAAGCCCAAAGCAGTGA |
| *SPTB* | AAGTGATCCAAGGCCAGACG | GTGATGAGCACACCTCCCAA |
| *FAM83E* | TGGAGGCTCTGTTGAGCAAG | GGTTGTAGGCACCTGAGTCC |
| *HGS* | AGACACCTTCACTTGGGTGC | CAGTCAGGACCACACGCTTC |
| *DSP* | TGCTCCCAGGTTAGGCAAAG | GATTCCCCAAGCCACACTGA |
| *TNNC1* | CAGAGGCTTGGTCCCTCTTG | AGAGGCCAGGGTAGGTACTG |
| *UBE2J1* | CAATCATTCTTGGTAGTGTTGCCCTTAGTC | GTGAGACAAGGAACTTTGACTTCTAGTCCC |
| *TYK2* | GCATACCCCAACCCTGTCCAATAAATGTGG | GATGGGGCCCAGCTGCTGCTTGCACACC |
| *ROBO4* | GCCCAGTAGACCTGTCTGCCTCCTTTTAG | CACCTGTGTTTTAGGATGGCATCCTCACT |
| *MYO15A* | AGATGGGCAGCCATGGGAAGCTGTGAGTT | GCCTGAGACACCTCCAGGGCAGGAATGTG |
| *HHLA1* | CTGAAGGTGCAGCTACCAGGGGAACTGC | ACCCATACCGGCTCATACAGATCTGTTAT |
| *TP53I3* | GTATGACCCACCTCCAGGAGCCAGCAAC | TTCTGCTGGGCGTCCGGTGTTAACATGTAC |
| *KIAA0100* | ATCCTGGGAGATATGGTTCAAGCTATTCCA | CTTGCTGTGCCTCAGCTGGCTGGTTCCAAT |
| *FCHSD1* | ACCCTGCAGAGCACACAGGTACAGCAACA | CTTGAGCTTTGTGCTTGTGACCATGCTCA |

**Supplementary Table S1. The sequences of the primers for off-target sites**
