# Supplementary material for: Targeted gene correction and functional recovery in achondroplasia patient-derived iPSCs
Source: Stem Cell Res Ther. 2021 Aug 28;12:485. doi: 10.1186/s13287-021-02555-8 (PMC8403427; doi:10.1186/s13287-021-02555-8)
Supplement: Supplementary file 3 — Additional file 3. Gene correction of ACH-iPSCs. [file 13287_2021_2555_MOESM3_ESM.pdf]

关于“CRISPR/Cas9 结合软骨发育不全（ACH）病人脂肪 MSCs 及其 iPSCs 对 ACH 治疗的研究”项目研究的伦理评审意见

“CRISPR/Cas9 结合软骨发育不全 (ACH) 病人脂肪 MSCs 及其 iPSCs 对 ACH 治疗的研究”项目属于基础研究，旨在研究治疗 ACH 的一种最新颖的方法，通过疼痛和创伤较小的抽脂技术获得 ACH 病人的脂肪组织，分离干细胞。故本项目有较大的临床应用前景。其研究成果有利于人类的健康事业。因在研究过程中将涉及到上海交通大学医学院附属仁济医院所收治的病人 / 病人脂肪组织标本。

经医学伦理委员会讨论，认为该研究符合临床实验研究的伦理道德规范和准则，特予批准其申报项目！研究过程中项目组须严格按照临床操作规范，并贯彻知情同意和保密原则，受试者 / 标本提供者应自愿签署知情同意书。

上海交通大学医学院附属仁济医院

医学伦理委员会

2015 年 3 月
